# Medical Men in the New Parliament

**Published:** 1910-02-19

**Authors:** 


The Hospital

A Journal of

The Medical Sciences and Hospital Administration.

New Series. No. 157, Vol. VI. [No. 1229, Vol. XLVII.] SATURDAY, FEBRUARY 19, 1910.

### Medical Men in the New Parliament.

Now that the elections are over, it is perhaps allowable for a purely professional paper, whose politics may be summed up by saying that its principal plank is the good of the profession, to comment upon the medical side of the struggle. The one incident, above all others, that has attracted the attention of the profession in this election has been the amount of capital made out of certain political posters issued in the Shoreditch-Hoxton constituency, where the winning candidate was Dr. Addison, who, as an anatomist, needs no introduction to our readers. It was alleged, with a great deal of unnecessary bitterness by some, that these posters, while maligning Dr. Addison, also aspersed the profession, and brought medical men into contempt by invoking passions that have slumbered since the days of Burke and Hare. As to the decency of political posters in general, it may be permissible to have more than one opinion; but so long as such things are allowed to be placarded in public it seems useless and, indeed, unreasonable to protest against a political opponent using every advantage, even that of the natural objection to dissection which is still held by the majority, to defeat his rival. Nor is it reasonable to suppose that party rancour should be softened because one of the rival candidates is a member of the medical profession. Dr. Addison, we suppose, solicited the votes of the Hoxton electors not because he is an expert anatomist, an examiner of the Royal College, or even a universally respected member of the profession, but because he has for years closely studied the political needs of that constituency in particular and of the country in general. He entered the struggle as a publicist, and as such had to expect to receive hard blows and caustic comments. In the circumstances it was unwise to have devoted so much time and trouble to condemning these absurd posters. We are glad to see Dr. Addison in Parliament, for we are convinced that, all party issues set aside, he will strive and work for the profession to which he belongs, but we cannot help feeling that his followers have acted foolishly in magnifying a specimen of electioneering ungentlemanliness into a general insult to medical men the world over.

Apart from Dr. Addison, the new Parliament will have the advantage of several other members of the profession in discussing questions in which the opinions of doctors might reasonably be supposed to be valuable to legislators. Sir William Collins, a veteran who has done very excellent work in the past, and who is not likely to be less energetic in the coming session, has been returned for his old constituency, St. Pancras. We do not doubt that Sir William's common-sense attitude on such matters as the Asylums Officers' Superannuation Bill, and his recognised ability in dealing with questions of medical interest have actively contributed towards his success. Sir Walter Foster, Sir G. H. Pollard, Sir R. J. Price, and Sir G. Scott Robertson among the English members, Dr. Rainy among the Scottish, and Drs. Dillon and Lynch among the Irish members, are also veterans of one or more sessions' standing whose return to their old battlefield everyone who has followed their Parliamentary careers and noticed how manfully they have borne their share of the work of legislation and how ably they have represented the profession in the House of Commons will cordially welcome. Dr. Alfred Hillier, who represents Eccles, and Dr. C. H. Dixon, who has been returned for Boston, both as members of the Opposition, are new men whose Parliamentary careers will be followed with interest. Dr. Hillier has done good work in the investigation of tuberculosis, and his advice on the legislative measures, which the near future may see evolved to deal with the prevention of phthisis, will be that of an expert, and, as such, doubly valuable, while his colonial experience should make his opinions on such matters as tropical hygiene of importance. Scotland returns one new member, Dr. Chapple, whose name is on the Register, and who, we believe, also hails from the colonies. Dr. Charles O'Neill, who has been returned for Armagh, is likewise a new member, and has written on anæsthetics. We sincerely trust that he will interest himself in the legislative measures with regard to this subject that have been outlined on more than one occasion, and received the general approval of the profession. The advisability of appointing a Royal Commission to investigate the whole subject of anæsthetics and the fatalities under them which have been so frequent of late, and which have drawn public attention so prominently to the need for legislative action, we have already pointed out, and we feel sure that Dr. O'Neill's special experience will make his active support on this question in Parliament an influential factor in passing any measure such as that of Dr. Hewitt, which may be brought forward during the coming session. There are many outstanding questions of great national interest that urgently demand the attention of Parliament. Unfortunately it is to be anticipated that these will receive but scanty consideration in the ensuing session, owing to the terrific party issues; but in their discussion, which must come some day, medical opinion should have a voice. For that reason we welcome the return of members of the profession to Parliament, believing as we do that it is in the public interest not only that the profession should be represented in the legislature, but that it should give the lay portion of that assembly the benefit of its experience in the discussion of questions which affect the public health and physical well being.

